# Low-Cost Unattended Design of Miniaturized 4 × 4 Butler Matrices with Nonstandard Phase Differences

**DOI:** 10.3390/s21030851

**Published:** 2021-01-27

**Authors:** Adrian Bekasiewicz, Slawomir Koziel

**Affiliations:** 1 Faculty of Electronics, Telecommunications and Informatics, Gdansk University of Technology, 80-233 Gdansk, Poland; koziel@ru.is; 2 Engineering Optimization & Modeling Center, Reykjavik University, 101 Reykjavik, Iceland

**Keywords:** Butler matrix, circuit miniaturization, design automation, numerical optimization, Internet of Things, 5G technology

## Abstract

Design of Butler matrices dedicated to Internet of Things and 5th generation (5G) mobile systems—where small size and high performance are of primary concern—is a challenging task that often exceeds capabilities of conventional techniques. Lack of appropriate, unified design approaches is a serious bottleneck for the development of Butler structures for contemporary applications. In this work, a low-cost bottom-up procedure for rigorous and unattended design of miniaturized 4 × 4 Butler matrices is proposed. The presented approach exploits numerical algorithms (governed by a set of suitable objective functions) to control synthesis, implementation, optimization, and fine-tuning of the structure and its individual building blocks. The framework is demonstrated using two miniaturized matrices with nonstandard output-port phase differences. Numerical results indicate that the computational cost of the design process using the presented framework is over 80% lower compared to the conventional approach. The footprints of optimized matrices are only 696 and 767 mm^2^, respectively. Small size and operation frequency of around 2.6 GHz make the circuits of potential use for mobile devices dedicated to work within a sub-6 GHz 5G spectrum. Both structures have been benchmarked against the state-of-the-art designs from the literature in terms of performance and size. Measurements of the fabricated Butler matrix prototype are also provided.

## 1. Introduction

Antenna arrays are the key components of modern communication devices. Their popular applications include long-term evolution and 5th generation (5G) cellular technology, where good performance is crucial for sustaining high data-transfer rates. Apart from the radiators, feeding network is an integral component of the antenna array. Its role is to provide appropriate excitation of the radiators (both magnitude- and phase-wise) so as to ensure the desired beamforming capability of the array [1,2,3,4,5,6].

Common array feeding realizations include variants of corporate [7,8,9] and series networks [10,11,12]. More complex structures are based on a combination of series–parallel feeds [13], networks with tunable power dividers [14], as well as structures dedicated to operating in mm-wave spectrum [15]. Butler matrices (BMs) and their derivatives belong to another class of feeding networks [16,17,18,19,20]. Their characteristic feature is the beamforming capability resulting from the ability to provide different phase shifts between the output ports depending on the selected input [17,20]. The conventional 4 × 4 (i.e., with four input and four output ports) BM—which offers output-port phase shifts of ±45° and ±135°, respectively—is a composite structure comprising four 90° hybrid couplers, with two crossovers and two 45° phase shifters (PSs) [21,22,23,24]. The circuit is characterized by large dimensions and lack of flexibility in terms of attainable phase differences. Consequently, the usefulness of standard BMs for modern mobile systems, including sensor networks and/or Internet of Things (IoT) devices interconnected using the 5G backbone, is limited. The following paragraphs contain more details concerning design of BM structures for contemporary applications.

In recent years, the design of Butler matrices for space-limited applications has gained significant attention of the research community [16,25,26,27,28,29,30,31,32,33]. Popular miniaturization techniques are oriented toward replacing BM building blocks with their miniaturized counterparts [29,30]. The latter are often implemented in the form of composite cells, representing appropriate combination of the high- and low-impedance transmission lines (TLs) [29,34]. However, the application of left/right-handed TLs for reduction of BM footprint has also been reported [28]. Miniaturization of conventional crossovers—realized as a composition of two branch line couplers (BLCs) interconnected through a 90° TL—is one of the leading approaches to design of small BMs [28,29,30]. Notwithstanding, substitution of conventional BM building blocks with their miniaturized counterparts results in modest miniaturization rates [28,30]. From this perspective, replacement of standard crossovers with alternative topologies featuring either small footprints or improved phase-related functionality seems to be an interesting and preferable alternative to compact BM design [19,35]. Other miniaturization methods depart from standard BM topologies. Instead, they eliminate certain matrix subcomponents or exploit multilayer substrates for structure implementation [26,27,32]. On the other hand, miniaturization resulting from application of the mentioned techniques is often insufficient to increase usefulness of BMs for space-limited systems [28,30]. Furthermore, the discussed compact topologies do not address the problem of improving BM performance.

The design of Butler matrices with increased flexibility in terms of achievable output-port phase differences has also been investigated in the literature [19,23,25,36]. The considered methods involve redesign of conventional structures, either through modification of their building blocks (i.e., couplers or delay lines) [19,23] or introduction of additional sub-circuits affecting BM performance [25,36]. In [23], enhanced control over beamforming capabilities has been achieved by replacing conventional 90° hybrid couplers by structures with adjustable phase shifts. In [19], similar effect has been achieved using PSs with unequal electrical lengths. The reasoning behind both methods is that improved control of phase can be achieved by increasing the number of relevant design parameters. An alternative technique, proposed in [36], boils down to enhancement of the conventional BM through connection of phase-reconfigurable TLs to its output ports. The mentioned methods proved to be useful for performance enhancement. However, they neglect the need of circuit miniaturization, which is important for application of matrices in contemporary systems.

The design solutions discussed above indicate that the difficulties related to improving BMs’ functionality and their miniaturization are perceived as separate problems. In other words, size-reduction strategies considered in the literature are dedicated to conventional topologies [28,29], whereas BM designs featuring improved performance suffer from large dimensions [23,36]. The development of BMs characterized by both small size and improved functionality is important for contemporary, space-limited applications such as IoT and sensor networks. Another problem is that the discussion of methods used to obtain specific solutions of miniaturized and/or performance-enhanced Butler matrices is often neglected in the literature. Instead, the emphasis is put on structure synthesis or analysis of the specific case study. In practice, however, synthesis (or generation of BM components) is just the beginning of the design procedure, which has to be followed by circuit assembly and careful adjustment of parameters oriented toward determination of the desired performance.

Due to high complexity, Butler matrix design is realized as a multistage process. The structure is first divided into individual building blocks, which are subject to a set of simplified design tasks oriented toward obtaining the desired performance [29]. Once the design parameters of all subcomponents are found, the matrix is assembled. The main benefit of such a strategy is that each step gradually approximates a satisfactory design solution. On the other hand, conventional design approaches involve time-consuming and repetitive analysis of the structure (and/or its building blocks). These often involve modifications of geometry parameters followed by visual inspection of structure responses [34,37]. Although the concept of semi-manual design proved to be fairly successful for circuits characterized by a relatively low number of variables and simple responses [37,38,39], its applicability to more complex problems, such as tuning of corporate feeds or BM topologies, is limited. One of the reasons is that the discussed structures are characterized by complex electrical responses that cannot be reliably tracked using manual techniques. From this perspective, Butler matrix design stages should include synthesis, development of BM components, and fine-tuning of the assembled circuit oriented toward maximization of performance. For the sake of reliability, each step should be controlled by appropriate algorithm and rigorously defined objective function. Yet another problem—rarely considered in the literature in terms of BM design [29]—is a large design cost associated with BM optimization. The latter stems from the necessity of using numerically expensive electromagnetic (EM) simulation models in order to obtain accurate responses of the matrix, and a large number of evaluations required by the optimization algorithm to converge.

Analysis of the available literature indicates that the design of BM structures for IoT and 5G applications is pertinent to several challenges. First of all, the development of portable electronics is associated with steadily increasing requirements concerning both miniaturization and performance. However, for BM structures, these problems are treated separately, which results in the development of either relatively small components with standard (or close-to standard) performance or bulky circuits with improved functionality [19,23,25,36]. Another problem involves lack of unified and well-established procedure—ranging from components synthesis to final tuning of the assembled circuit—dedicated to BM design. Moreover, conventional approaches to the development of BM structures are unreliable and inefficient due to a large number of figures representing matrix performance (all of which have to be accounted for at a time) [27,28,30]. Although numerical optimization seems to be appropriate for addressing the difficulties pertinent to parameter adjustments, it suffers from high cost related to a large number of CPU-heavy evaluations required by the algorithm to converge. Consequently, maintaining low computational budget—e.g., using surrogate methods [40,41,42]—is of high importance in increasing the usefulness of algorithm-based approaches to BM design. The motivation of this work is to address the discussed challenges pertinent to design of modern BM circuits.

In this work, a bottom-up procedure for low-cost unattended design of miniaturized 4 × 4 Butler matrices with nonstandard output-port phase differences has been presented. The proposed method consists of two main steps involving synthesis and optimization of BM building blocks, followed by two-stage EM-driven tuning of the assembled matrix. The main contributions of the work include: (i) development of an automated methodology for sequential design of BM matrix components; (ii) determination of rigorously defined objective functions that facilitate handling the components/matrix design using robust numerical optimization algorithms; (iii) integration of all BM design stages (i.e., components synthesis, implementation, integration, and EM-driven tuning of assembled matrix) into a design framework; and (iv) validation of the presented method using two compact BMs with nonstandard output-port phases. The considered case studies involve design of structures featuring phase shifts of {−30°, 150°, −120°, 60°} and {−20°, 160°, −110°, 70°}, respectively. The footprints of the optimized circuits are only 696 and 767 mm^2^. To the best of the authors’ knowledge, this is the first work that thoroughly discusses the problem concerning automated BM design oriented for both miniaturization and enhancement of electrical performance. According to benchmark results, the computational cost of circuit design using the presented framework is over 80% lower compared to more conventional strategy based on direct EM-driven optimization of the assembled matrix. The optimized structures have been compared against state-of-the-art circuits from the literature in terms of size and performance. A comparison of simulation and measurement results is also provided.

## 2. Design Problem and Models of Butler Matrix Components

The automated design process presented here is governed by numerical optimization algorithms. This section contains formulation of the design problem and definition of models used by the proposed framework. For the sake of consistency, a brief discussion of numerical algorithms used in the work is also included. The details concerning mechanisms embedded into the proposed design framework are provided in Section 3.

### 2.1. Design Problem

The design problem can be formulated as a nonlinear minimization task of the form [40]

(1) $$x^{*}=\arg\underset{x}{\min}U\left(R\left(x\right)\right)$$

where ***R***(***x***) is the response of the structure (be it the BM or its sub-circuit) under design. The vectors ***x*** and x^*^ represent adjustable parameters of the structure and the optimal design to be found, whereas *U* denotes an objective function. The goal of (1) is to find x^*^ through minimization of *U*. The latter “translates” the structure response into a scalar value, which is used by numerical algorithm to govern the design process toward the optimal solution. In other words, the function *U* represents the structure performance—calculated based on responses of the circuit—at the given design.

### 2.2. Models of Butler Matrix Components

**Figure 1:**
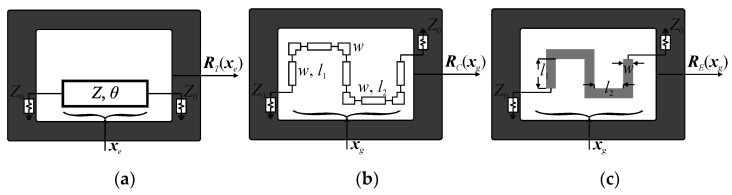
Illustration of structure representations (here, a phase shifter) supported by the design method: (**a**) ideal transmission line (TL) model ***R****_T_*(***x****_e_*) with ***x****_e_* = [*Z θ*]*^T^*, (**b**) equivalent-circuit model ***R****_C_*(***x****_g_*), and (**c**) high-fidelity electromagnetic (EM) model ***R****_E_*(***x****_g_*). The vector of geometry parameters ***x****_g_* = [*w l*_1_ *l*_2_]*^T^* is the same for ***R****_C_* and ***R****_E_* models.

In the proposed bottom-up design framework, the determination of the final BM geometry is preceded by synthesis, implementation, and optimization of its individual components. The responses of BM sub-circuits can be obtained through evaluation of the ideal transmission-line ***R****_T_*(***x****_e_*), equivalent-circuit ***R****_C_*(***x****_g_*), or electromagnetic ***R****_E_*(***x****_g_*) models, respectively. Here, ***x****_e_* denotes the electrical parameters (characteristic impedance and electrical length of the transmission line), whereas ***x****_g_* is the vector of geometry variables. Conceptual illustrations of discussed structure representations are shown in Figure 1. It should be emphasized that the type of structure model matches the complexity of design task and size of the search space in terms of computational cost and accuracy. In other words, the model fidelity increases along with narrowing the search space to the region of interest. Here, the synthesis and correction/refinement of BM components at the level of their electrical parameters is realized using ***R****_T_* model, whereas ***R****_C_* and ***R****_E_* are applied for miniaturization-oriented development of building-blocks topologies and to obtain accurate responses for optimization and fine-tuning of the components.

### 2.3. Butler Matrix Representations

**Figure 2:**
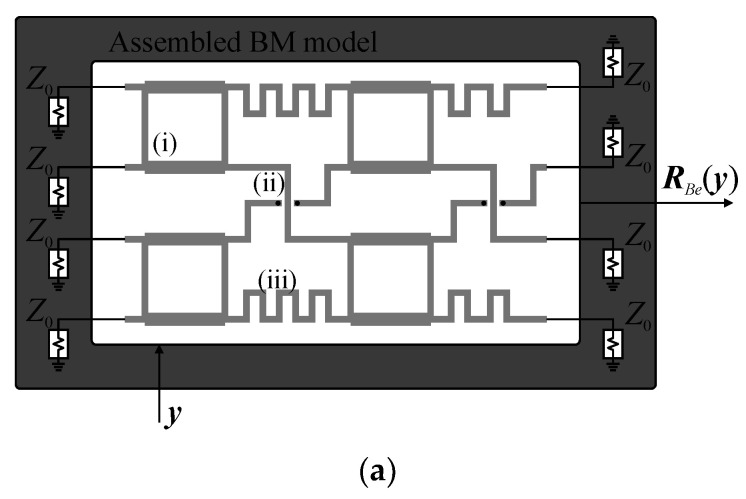

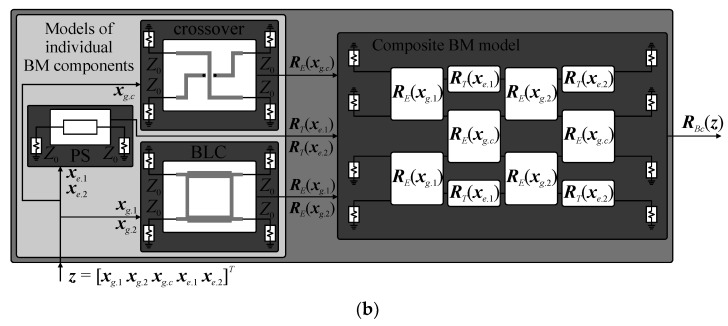
A conceptual illustration of the Butler matrix (BM) models: (**a**) high-fidelity EM model of the assembled matrix where (i)–(iii) denote branch line coupler (BLC), crossover, and phase-shifter, respectively, and (**b**) a composite representation of the structure. Frequency characteristics of the composite BM are obtained from the responses of individual components. In this illustration, ***R****_Bc_* is calculated using EM model responses of crossover (design ***x****_g.c_*) and BLCs (two designs: ***x****_g_*_.1_ and ***x****_g_*_.2_), as well as ideal TL model of phase shifters (two designs: ***x****_e_*_.1_ and ***x****_e_*_.2_).

**Figure 3:**
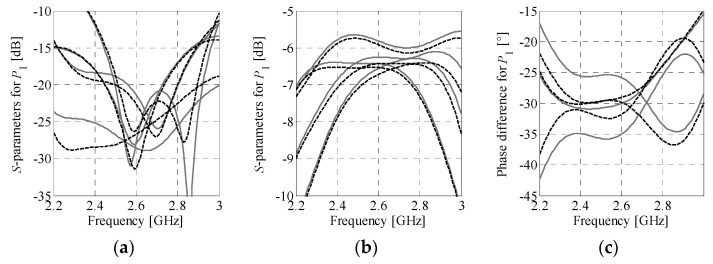
Frequency characteristics of the composite (solid gray) and assembled (dashed black) BM models at some design: (**a**) reflection and isolation, (**b**) transmission, and (**c**) phase difference between output ports. The responses are obtained for excitation of both structures through the first input port.

The Butler matrix models used here are defined as follows. Let ***R****_Be_*(***y***) and ***R****_Bc_*(***z***) denote the high-fidelity EM model of the assembled BM (i.e., the one where all BM subcomponents are implemented and physically interconnected in the form of a single full-wave EM design) and the composite representation of the structure, respectively. The variables ***y*** and ***z*** represent their design parameters. The composite model responses are obtained using transmission-line theory from the *S*-parameter characteristics of individual BM subcircuits [43,44]. A conceptual illustration of the ***R****_Be_* and ***R****_Bc_* models is shown in Figure 2. It should be noted that evaluation of the ***R****_Bc_* model is to be preceded by simulations of its subcomponents (cf. Figure 2b). Nonetheless, the composite model is characterized by much lower evaluation cost compared to ***R****_Be_*. The vector ***z*** can be set equal to ***y*** but can also represent a composition of electrical and/or geometry variables of the sub-circuits selected for optimization (cf. Figure 2b). Example responses obtained from evaluation of ***R****_Be_* and ***R****_Bc_* models for the ***z*** = ***y*** are shown in Figure 3. Despite reduced accuracy w.r.t. ***R****_Be_* (especially phase-wise), resulting from neglecting the coupling between adjacent components and loss at their interconnection, the composite model is useful for narrowing the search space to the region of interest before the fine-tuning stage.

### 2.4. Optimization Algorithms

As already mentioned, the proposed design method is governed by numerical optimization algorithms. The framework presented here exploits two routines: a trust-region-based gradient method and an unconstrained variant of the bisection algorithm. To make the work self-contained, both routines are briefly discussed below.

#### 2.4.1. Trust-Region-Based Optimization

The main optimization engine is a gradient algorithm embedded in a trust-region framework. The method generates a series of approximations (*i* = 1, 2, 3, …) to the final design by solving [45]

(2) $$x^{\left(i+1\right)}=\arg\underset{x:\left\|x-x^{\left(i\right)}\right\|\leq r^{\left(i\right)}}{\min}U\left(G^{\left(i\right)}\left(x\right)\right)$$

where ***G***^(*i*)^ is the first-order Taylor surrogate constructed from the *S*-parameter responses of the structure at hand. The model is given as [45]

(3) $$G^{\left(i\right)}\left(x\right)=R\left(x^{\left(i\right)}\right)+J\left(x^{\left(i\right)}\right)\cdot \left(x-x^{\left(i\right)}\right)$$

Here, ***R***(***x***^(*i*)^) is the response of the structure at hand. The perturbations for generation of the Jacobian ***J*** are obtained using a large-step finite differentiation [45]. Note that the model ***G***^(*i*)^ may be constructed using a combination of the responses determined from evaluations of the low- and high-fidelity representations of the circuit under design [46]. The parameter *r*^(*i*)^ represents the trust-region radius, which is iteratively updated based on the calculated gain ratio, i.e., the obtained versus predicted change of the objective function. The radius is updated using standard rules [45]. A more detailed discussion of the algorithm can be found in [45,46].

#### 2.4.2. Bisection-Based Heuristic

Another algorithm used in this work is a simple unconstrained variant of the bisection method [47]. Let ***x***_0.1_ and ***x***_0.2_ represent the starting points for the algorithm. Here, the vector ***x***_0.1_ is obtained as a result of structure synthesis, whereas ***x***_0.2_ represents a perturbation of all design parameters w.r.t. ***x***_0.1_. The algorithm flow is as follows:

1. Set *i* = 0, ***x***_0.1_, and ***x***_0.2_; set Δ***x*** = |***x***_0.1_ − ***x***_0.2_|; set ***x***^(0)^ = 0.5(***x***_0.1_ + ***x***_0.2_).
2. Generate interval [***x***_1_^(*i*)^, ***x***_2_^(*i*)^] around the point ***x***^(*i*)^, where ***x***_1_^(*i*)^ = ***x***^(*i*)^ − αΔ***x*** and ***x***_2_^(*i*)^ = ***x***^(*i*)^ + αΔ***x***.
3. If *U*(***x***_1_^(*i*)^)*U*(***x***_2_^(*i*)^) ≥ 0, set $x^{\left(i+1\right)}=\{\begin{array}{c} x_{1}{}^{\left(i\right)}-\alpha \Delta x^{\left(i\right)}\;\;\mathrm{when}\;U(x_{1}{}^{\left(i\right)})>0\\ x_{2}{}^{\left(i\right)}+\alpha \Delta x^{\left(i\right)}\;\;\mathrm{when}\;U(x_{1}{}^{\left(i\right)})<0 \end{array}$, *i* = *i* + 1 and go to step 2; otherwise go to step 4.
4. Set ***x***^(*i*+1)^ = 0.5(***x***_1_^(*i*)^ + ***x***_2_^(*i*)^), ***x***_1_^(*i*+1)^ = ***x***_1_^(*i*)^, ***x***_2_^(*i*+1)^ = ***x***_2_^(*i*)^, *i* = *i* + 1.
5. If *U*(***x***_1_^(*i*)^)*U*(***x***^(*i*)^) < 0, set ***x***_2_^(*i*)^ = ***x***^(*i*)^ and go to step 7; otherwise go to step 6.
6. If *U*(***x***^(*i*)^)*U*(***x***_2_^(*i*)^) < 0, set ***x***_1_^(*i*)^ = ***x***^(*i*)^ and go to step 7.
7. If |***x***^(*i*)^ − ***x***^(*i*−1)^| ≤ *ε*, set x^*^ = ***x***^(*i*)^ and END; otherwise go to step 4.

It should be noted that *α* and *ε* are user-defined parameters. Here, *U*(***x***) = *U*(***R***(***x***)) represents the objective function value calculated based on the model response at the design ***x***. The discussed bisection-based algorithm is useful for approximating dimensions of the miniaturized BM components.

## 3. Methodology

The framework presented involves two main design stages: (i) synthesis and design of individual BM components and (ii) optimization of the composite BM model followed by fine-tuning of the assembled structure. Here, a detailed discussion of each design step and a summary of the presented methodology are provided. The numerical and experimental validation of the framework is considered in Section 4.3.

### 3.1. Synthesis of Butler Matrix and Its Components

**Figure 4:**
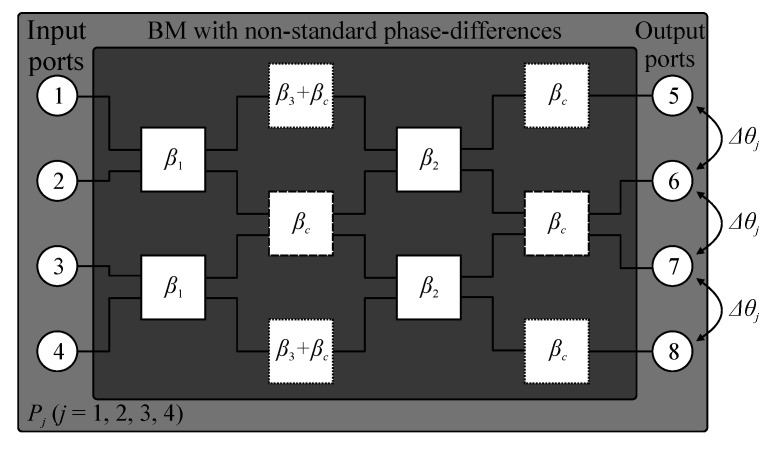
Butler matrix with nonstandard output-port phase differences: a conceptual illustration.

A conceptual illustration of the considered 4 × 4 Butler matrix with nonstandard output-port phase differences is shown in Figure 4. The structure comprises two pairs of hybrid branch line couplers with adjustable phase, as well as two crossovers and four phase shifters. Excitation of the matrix through the selected input port *P_j_* (*j* = 1, 2, 3, 4) allows for obtaining the phase differences Δ*θ_j_* between its output ports *P*_5–8_. The relation between output phase shifts and electrical lengths of the structure components is given as [23]

(4) $$\beta_{1}=0.5\beta_{2}-0.25\pi$$

(5) $$\beta_{2}=2\Delta \theta_{1}$$

(6) $$\beta_{3}=-0.25\pi$$

**Figure 5:**
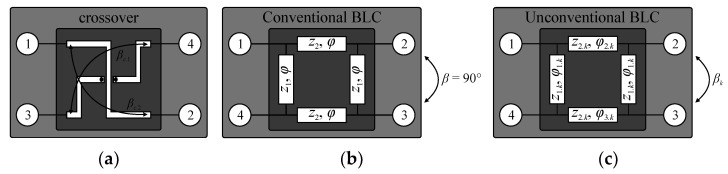
Crossover and hybrid BLCs configurations: (**a**) crossover structure with highlight on phase shifts between crossed TLs (desired phase shift of the crossover is *β_c_* = *β_c_*_.1_ = *β_c_*_.2_), (**b**) conventional BLC with *z*_1_ = 0.707, *z*_2_ = 1, and *φ* = 90° (phase shift: *β* = 90°), and (**c**) modified BLC circuit with extra degrees of freedom *φ*_2*.k*_, *φ*_3*.k*_ w.r.t. conventional design that enables adjustment of phase shift *β_k_* (the parameters *z*_1_ and *z*_2_ are normalized w.r.t. *Z*_0_ = 50 Ω).

Here, *β*_1_ and *β*_2_ represent phase shifts introduced by the first and second pair of BLCs, whereas *β*_3_ is the electrical length of the phase shifter. As shown in Figure 4 and Figure 5a, the component *β_c_* represents the electrical length of crossovers. Based on (4)–(6), one can infer that phase differences at output ports of the BM are a function of *β*_2_. Consequently, Δ*θ*_1_ = 0.5*β*_2_, Δ*θ*_2_ = 0.5*β*_2_ + *π*, Δ*θ*_3_ = 0.5*β*_2_ − 0.5*π*, and Δ*θ*_4_ = 0.5*β*_2_ + 0.5*π* [23]. The feasible ranges of phase shifts *β*_1_ and *β*_2_—i.e., the ones for which realizable topologies of compact BLCs can be obtained—vary from −30° to −90°. Therefore, the attainable output-port phase differences are Δ*θ*_1_ ∈ [−45°; −15°], Δ*θ*_2_ ∈ [135°; 165°], Δ*θ*_3_ ∈ [−135°; −105°], and Δ*θ*_4_ ∈ [45°; 75°], respectively. The parameters *β*_1–3_ are used as the starting points for design of individual matrix components.

The illustration of the BLC structure capable of obtaining the desired phase and its comparison with conventional 90° hybrid is shown in Figure 5b,c. Given the phase difference *β_k_* (*k* = 1,2), *k*th coupler consists of a pair of 90° TL sections with characteristic impedance *z*_1*.k*_ and equal electrical length of *φ*_1.*k*_, as well as a pair of TLs with impedance *z*_2.*k*_ and electrical lengths *φ*_2.*k*_ and *φ*_3.*k*_, respectively. Electrical parameters of the circuit can be obtained from the following equations [48]:

(7) $$z_{1.k}=\sqrt{\frac{z_{2.k}^{2}}{1-z_{2.k}^{2}}}$$

(8) $$z_{2.k}=\frac{\tan\left(\beta_{k}\right)\left(\tan^{2}\left(0.5\varphi_{2.k}\right)-1\right)}{2\tan\left(0.5\varphi_{2.k}\right)}$$

(9) $$\varphi_{3.k}={2\tan}^{-1}\left(\sqrt{\frac{1}{\tan\left(0.5\varphi_{2.k}\right)}}\right)$$

Here, (8) is solved for *φ*_2.*k*_ with *z*_2.*k*_ = 0.5 × 2^0.5^ to provide closed-form estimation of BLC parameters:

(10) $$\varphi_{2.k}=\pi -\tan^{-1}\left(\sqrt{2}\tan\left(\beta_{k}\right)\right)$$

Although solving (7), (9), and (10) using the considered *z*_2.*k*_ provides good estimation of the *φ*_2.*k*_ and *φ*_3.*k*_ w.r.t. the required phase shift, it does introduce power split error at the operating frequency. To address the problem, the ideal model of the coupler is optimized using algorithm of Section 2.4.1. The objective function is

(11) $$U_{1}=\alpha_{1}\Delta C^{2}+\alpha_{2}{\left(\frac{\max\left(M-M_{0},0\right)}{M_{0}}\right)}^{2}+\alpha_{3}\left({\left(\phi_{1}-\phi_{0.1}\right)}^{2}+{\left(\phi_{2}-\phi_{0.2}\right)}^{2}\right)$$

Here, (11) is minimized based on the ***R****_T_*(***x****_e_*) model responses, where ***x****_e_* = ***x****_e.k_* = [*z*_1.*k*_ *z*_2.*k*_ *φ*_2.*k*_ *φ*_3.*k*_]*^T^* represents the vector of electrical parameters of *k*th BLC (note that *φ*_1.*k*_ = 90°). The parameter Δ*C* = ||*S*_31_| − |*S*_21_|| denotes the power-split imbalance at the center frequency *f*_0_, whereas *M* = max(|*S*_11_|, |*S*_41_|) is an in-band performance of the BLC over the frequency range of interest defined around *f*_0_ [46]. The figures *φ*_1_ = ∠(*S*_21_/*S*_31_) and *φ*_2_ = ∠(*S*_24_/*S*_34_) are phase shifts at *f*_0_. The parameters *M*_0_ = −20 dB, *φ*_0.1_ = *β_k_*, and *φ*_0.2_ = *β_k_* − π represent target values. The scaling coefficients [*α*_1_ *α*_2_ *α*_3_] = [1000 500 1000] are determined so as to ensure balanced contribution of the design requirements to the aggregated objective function (11), [49,50]. In other words, they maintain similar relative importance of sub-elements in (11) during the optimization process. The selected values are appropriate for BM circuit components synthesized using (7)–(10).

### 3.2. Sequential Design of BM Components

The design of individual BM components is realized as a sequential process that involves determination of BLCs and crossovers geometries, as well as by tuning of the phase shifters. For the given center frequency *f*_0_, the crossover—see Figure 5a for illustration—can be considered as a “static” component of the matrix. In other words, its dimensions do not change when the BM is redesigned for another set of output phase differences. Consequently, the same design can be used to realize a range of Δ*θ_j_*. The crossover dimensions are adjusted in two steps. First, the selected geometry is optimized for minimization of *U*_2.1_ = max(|*S*_11_|, |*S*_33_|) at the center frequency *f*_0_, where |*S*_11_|, |*S*_33_| represent reflection of the crossed TLs. Next, the design is oriented toward ensuring that electrical lengths of the crossed transmission lines *β_c_*_.1_ and *β_c_*_.2_ are equal at *f*_0_. This is achieved by minimization of the objective function *U*_2.2_ = (*β_c_*_.1_ − *β_c_*_.2_)^2^. In each design step, the optimization is carried out using the algorithm of Section 2.4.1 and the ***R****_E_*(***x****_g.c_*) model responses. Here, the vector ***x****_g.c_* represents geometry parameters of the crossover. The final design ***x***_g.c_^*^ structure provides equal length lines (*β_c_ = β_c_*_.1_ ≈ *β_c_*_.2_) with low reflection and high isolation levels (note that the term isolation refers to attenuation of the signal and is expressed as an absolute value of the transmission—in dB—between the selected pair of ports), all important for high BM performance.

The design stage involves development of compact BLCs. For each coupler, the initial design is synthesized as described in Section 3.1. The design of *k*th BLC (cf. Section 3.1) can be summarized as follows:

1. Decompose ideal BLC model to individual TLs.
2. Use electrical parameters of TLs as the reference for development of miniaturized BLC sections.
3. Optimize the BLC sections to match electrical parameters of the reference TLs.
4. Construct miniaturized BLC using the optimized cells and define the vector of its design parameters ***x****_g.k_*.
5. Optimize compact BLC using objective function (11) and algorithm of Section 2.4.1.

Note that, in step 3, each section of the miniaturized BLC must be optimized to ensure geometrical consistency of the structure, as well as to provide sufficient flexibility for the tuning of phase shifts. In steps 2, 4, and 5, the BLC optimization is carried out using the algorithm of Section 2.4.1, whereas in step 3 the routine of Section 2.4.2 is used. It should be emphasized that ***R****_E_* model evaluations are used only in the last stage of the BLC development, whereas models ***R****_T_* and ***R****_C_* are used in stage 1 and stages 2–4, respectively. Shifting the optimization burden to the simplified models is important for maintaining low cost of coupler development. For more detailed discussion on design of miniaturized BLCs, see [29,34,46,51]. The optimized high-fidelity BLC designs ***x****_g_*_.1_^*^ and ***x****_g_*_.2_^*^ are used as the starting point for BM tuning.

The final stage of the sequential design process involves development of phase shifters. Conventional BM structures comprise equal-length PSs. Here, however, unequal-length shifters are used to increase flexibility of the BM in terms of control over the output-port phase differences. The electrical parameters of PSs are optimized using the composite BM model. In this step, the model integrates EM responses of optimized BLCs and crossovers (which remain fixed in the optimization process). The phase shifters are implemented in the form of ideal TLs. The composite model is optimized to minimize the following function:

(12) $$\begin{array}{l} U_{3}=\alpha_{1}{\left(\frac{\max\left(M_{B}-M_{B0},0\right)}{M_{B0}}\right)}^{2}+\alpha_{2}{\left(\frac{\max\left(\Delta C_{B}-\Delta C_{0},0\right)}{\Delta C_{0}}\right)}^{2}+\\ \;\;\;\;\;\;\;+\alpha_{3}{\left(\frac{\max\left(P_{B}-P_{0},0\right)}{P_{0}}\right)}^{2}+\alpha_{4}{\left(\frac{\max\left(M_{Bf0}-M_{f0},0\right)}{M_{f0}}\right)}^{2} \end{array}$$

where

(13) $$\Delta C_{B}=\frac{1}{N}\sum_{1}^{N}\left|\max\left(\Delta C_{E.j}\right)-\min\left(\Delta C_{E.j}\right)\right|$$

(14) $$P_{B}=\frac{1}{N}\sum_{1}^{N}\left|\max\left(P_{E.j}\right)-\min\left(P_{E.j}\right)\right|$$

Here, *M_B_* and *M_Bf_*_0_ represent the maximum value of the reflection and isolation responses between BM input ports (expressed in dB) within the frequency range of interest and at *f*_0_, respectively. The figure Δ*C_E.j_* denotes the magnitude (in dB) of transmission to the output ports at *f*_0_ when the structure is fed through the *j*th port (*N* = 4). Similarly, *P_E.j_* is a normalized phase difference at the output ports for excitation through *j*th port. The parameters *M_B_*_0_ = −15 dB, Δ*C*_0_ = 0.2 dB, *P*_0_ = 1.5°, and *M_f_*_0_ = −30 dB represent the target values for matrix design. The weights [*α*_1_ *α*_2_ *α*_3_ *α*_4_] = [400 1 1 10] are determined based on numerical studies.

The optimized electrical lengths of PSs are used as the target for determination of each shifter’s physical dimensions. The phase shifters are implemented in the form of meandered TLs. The optimization of each PS is realized separately and is oriented toward minimization of (12). The initial dimensions of the meander lines are determined based on the transmission-line theory. The design objective for refinement of *p*th (*p* = 1, 2, 3, 4) phase shifter geometry is *U*_4_ = (*e*_0*.p*_ − *e_p_*)^2^, where *e_p_* is the electrical length of the structure under design and *e*_0*.p*_ represents the target length. Due to low evaluation cost and quick convergence of the algorithm (2), each meandered PS is implemented only in the form of high-fidelity model ***R****_E_*. The Butler matrix components determined in this stage of the design process are used for further optimization and tuning of the assembled structure.

### 3.3. Butler Matrix Optimization and Fine-Tuning

For the sake of low computational cost, the BM optimization is realized using the composite model ***R****_Bc_*. Here, the parameters of all BLCs and PSs are enabled for adjustment. The optimization is oriented toward minimization of (12) using algorithm of Section 2.4.1. The main goal of this step is to further narrow down the search space to the region of interest so as to reduce the number of ***R****_Be_* evaluations required to find the final design. It should be reiterated that the evaluation cost of ***R****_Be_* is much higher (at least 3-fold) compared to the simulation cost of the composite structure.

Fine-tuning of the structure is again realized through minimization of (12). Here, the low cost of the process is maintained using a modified Taylor-expansion model (3), which exploits Jacobian ***J*** constructed based on simulations of the composite model, whereas the high-fidelity model simulations are performed only at the center design (i.e., ***R***(***x***^(i)^) = ***R****_Be_*(***y***^(i)^)) [46]. Consequently, each iteration of the tuning process requires only two EM simulations of each subcomponent (i.e., a total of four and eight simulations for couplers and phase shifters, respectively) and single evaluation of the assembled BM. The design y^*^ obtained after the fine-tuning stage is the final solution of the presented design process.

### 3.4. Summary of the Design Framework

The proposed framework for design of miniaturized Butler matrices with unconventional phase differences can be summarized as follows:

1. Define the desired performance of the Butler matrix.
2. Perform synthesis of the BM structure using (4)–(6).
3. Perform fine-tuning of the crossover to match length of its TLs and extract β_c_ (cf. Section 3.2).
4. Use (7), (9), (10) to synthesize the BLCs.
5. Adjust performance of synthesized BLCs through minimization of (11).
6. Set k = 1.
7. Perform topology development of kth miniaturized BLC and optimize its EM model.
8. If k = 2, go to step 9; otherwise set k = k + 1 and go to step 7.
9. Optimize ideal models of phase shifters through minimization of (12), set p = 1.
10. Generate initial dimensions of pth PS and optimize its EM model (cf. Section 3.2).
11. If p = 4, go to step 12; otherwise set p = p + 1 and go to step 10.
12. Optimize composite model of the BM.
13. Perform fine-tuning of the BM.

It should be noted that the design process described here is automated. Consequently, once the models of components and assembled BM are prepared, the role of the user is reduced only to definition of the performance requirements. The design bounds for constrained optimization stages (i.e., the ones governed by the TR algorithm) are defined as ±30% around the starting point for each step. The computational cost of the design process realized using the proposed method is comparable to around a dozen of ***R****_Be_* model simulations. Typically, each step that involves EM model evaluations requires no more than 10 iterations of the algorithm (2) to converge. The cost associated with synthesis of BM, BLCs, and PSs is negligible as it only requires evaluations of the transmission line or equivalent-circuit models. It is worth mentioning that conventional matrix with phase shifts of ±45° and ±135° is just a special case for the presented methodology. Consequently, the proposed framework is applicable to the design of eight port BMs, for which the requirements concern size reduction, performance enhancement, or combination of thereof. From this perspective, the methodology represents a generalized approach to design of 4 × 4 Butler matrices discussed in Section 3.

## 4. Numerical Results and Experiment

In this section, the proposed bottom-up design framework is demonstrated based on two examples of a compact 4 × 4 Butler matrices. Both structures are implemented on a dielectric substrate with *ε_r_* = 3.48, *h* = 0.168 mm, and tan *δ* = 0.0037. The center frequency for the considered BMs is set to *f*_0_ = 2.6 GHz, whereas the desired operational bandwidth is from 2.5 to 2.7 GHz. The considered range covers sub-6 GHz bands used by the 5G technology. The presented framework has been benchmarked against conventional approach to BM design. Furthermore, the considered matrices have been compared against the state-of-the-art designs from the literature. The measurement results obtained for one of the matrices have also been included and discussed.

The presented framework is implemented in MATLAB. The latter controls synthesis of BM components, integration of circuits, optimization, as well as bidirectional communication with external simulation packages. Evaluations of the equivalent-circuit models are performed using Keysight ADS software, whereas simulations of the EM models are handled using CST Studio packages.

### 4.1. Butler Matrix 1

The first design example is a Butler matrix featuring an output-port phase shifts of Δ*θ*_1–4_ = {−30°, 150°, −120°, 60°}. The electrical parameters of the structure components (cf. Section 3.1), i.e., *β*_1_ = −75°, *β*_2_ = −60°, and *β*_3_ = −45°, have been synthesized from (4)*–*(6).

**Figure 6:**
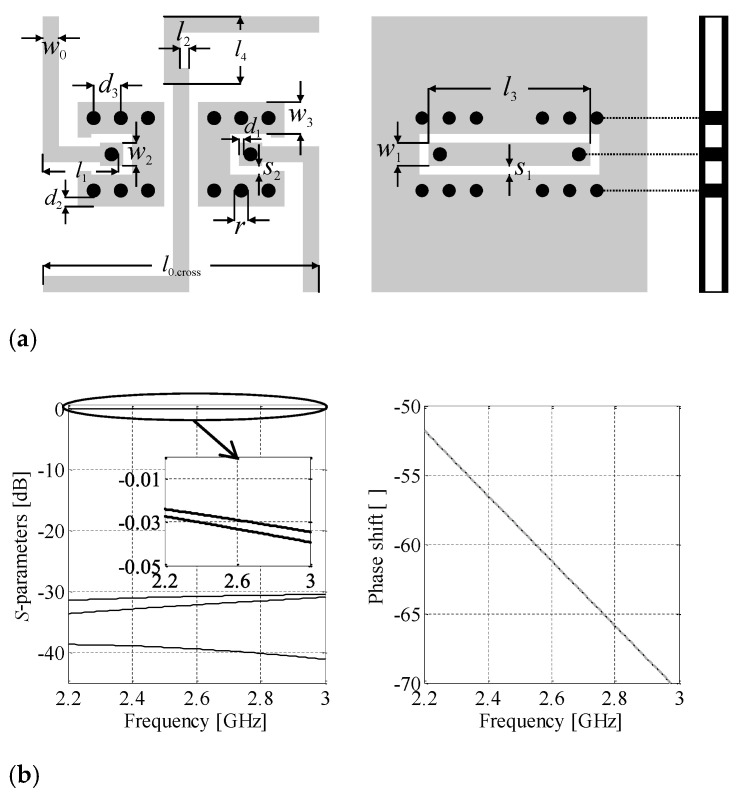
Compact crossover: (**a**) geometry of the structure, as well as (**b**) magnitude and phase characteristics of the circuit obtained based on EM simulations. Note that the electrical lengths of crossed lines (solid gray and dashed black) are virtually the same.

In order to maintain small BM dimensions, the crossover used here departs from the standard designs composed as a cascade connection of hybrid couplers [28,29,33]. Instead, it is based on a microstrip-to-coplanar-waveguide (CPW) transition. The design is characterized by small dimensions, as well as good isolation between crossed TLs [52]. The crossover topology is shown in Figure 6a. The structure introduces a meander to one of the transmission lines which can be adjusted to match electrical length of the crossed TLs. The design parameters of the component are ***x****_g.c_* = [*l*_1_ *l*_2_ *w*_1_ *s*_1_ *s*_2_ *d*_1_ *d*_2_ *d*_3_]*^T^*. The relative dimensions are *w*_2_ = 2*d*_1_ + *r*, *w*_3_ = 2*d*_2_ + *r*, *l*_0.cross_ = 2*l*_1_ + *w*_0_ + 2(*d*_1_ + 2*d*_2_ + 3*r* + 2*s*_2_), *l*_3_ = *w*_0_ + 2(4*r* + 2*s*_2_ + 2*d*_1_ +2*d*_2_), *l*_4_ = 0.5*w*_1_ + *s*_1_ + 2*r* + 2*d*_2_ − 2*s*_2_, whereas *r* = 0.3. The parameter *w*_0_ = 0.35 to ensure 50 Ω input impedance. Note that the unit for all dimensions is “mm.” The initial design ***x****_g.c_*^(0)^ = [1.5 0 0.3 0.2 0.2 0.1 0.15 0.6]*^T^* has been obtained in the course of structure development. The final design ***x***_g.c_^*^ = [1.5 0.2 0.49 0.2 0.2 0.1 0.2 0.59]*^T^* has been found through minimization of the objective function defined in Section 3.2. The optimized structure is characterized by a small footprint of only 6.05 mm × 6.05 mm = 36.6 mm^2^. Furthermore, it offers reflection below −30.5 dB, as well as transmission and isolation of around −0.03 and 39.5 dB (all at the center frequency), respectively. Owing to the use of meander lines, the difference of electrical length between crossed lines is kept below 0.02°, whereas the phase shift introduced by the structure amounts to *β_c_* = −61.2° (cf. Section 3.2). The magnitude and phase characteristics of the optimized component are shown in Figure 6b.

**Figure 7:**
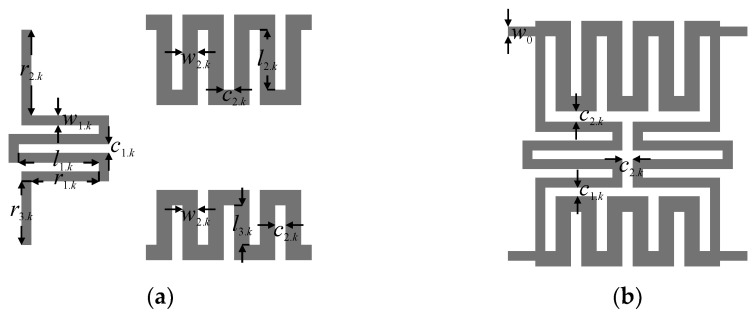
Compact BLC with flexible phase difference: (**a**) individual sections of the structure and (**b**) geometry of the assembled circuit.

**Figure 8:**
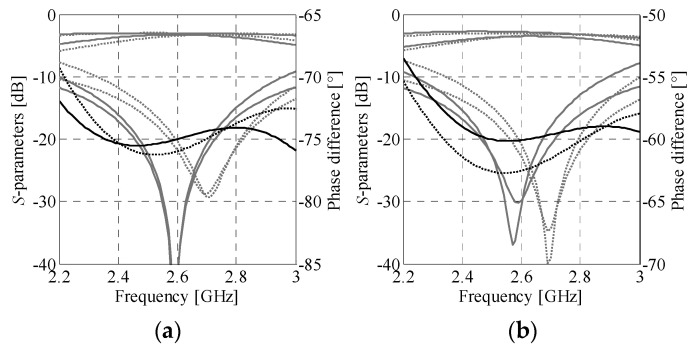
EM model responses of miniaturized BLC structures – magnitude (gray) and phase (black) at the initial (····) and optimized (––) designs: (**a**) BLC_1_ (realized phase difference *β_1_* = −75°) and (**b**) BLC_2_ (realized phase difference *β*_2_ = −60°).

Next, the design of individual BLC structures has been performed. The initial parameters of the first BLC *Z*_1.1_ = 35.35 Ω, *φ*_2.1_ = 100.73°, *φ*_3.1_ = 79.27° and the second coupler *Z*_1.2_ = 35.35 Ω, *φ*_2.2_ = 112.21°, *φ*_3.2_ = 67.79° have been obtained from (7), (9), and (10). Note that *Z*_1–2.*k*_ = *Z*_0_·*z*_1–2.*k*_ (here, *z*_2.*k*_ = 0.5·2^0.5^ and *φ*_1–2.*k*_ = 90°). The corrected parameters, obtained through optimization of ideal models (cf. Section 3.1), are *Z*_1.1_ = 44.65 Ω, *Z*_2.1_ = 32.16 Ω, *φ*_2.1_ = 100.56°, *φ*_3.1_ = 79.44° for BLC_1_ and *Z*_1.2_ = 42.59 Ω, *Z*_2.2_ = 32.20 Ω, *φ*_2.2_ = 110.71°, *φ*_3.2_ = 69.30° for BLC_2_. The obtained electrical parameters have been used as the initial designs for microstrip-line-based implementations of the miniaturized BLCs. The geometry of the compact BLC structure with highlight on its individual transmission line sections is shown in Figure 7. The vector of *k*th coupler design parameters is ***x****_g.k_* = [*w*_1.*k*_ *w*_2.*k*_ *c*_1.*k*_ *c*_2.*k*_ *l*_1.*k*_ *l*_2.*k*_ *l*_3.*k*_]*^T^*. The dimensions of each section have been recalculated from electrical parameters using the transmission line theory and adjusted using the bisection-based algorithm of Section 2.4.2 assuming *c*_1.*k*_ = *c*_2.*k*_ = 0.3 mm. Then, the obtained initial designs ***x****_g_*_.1_^(0)^ = [0.39 0.58 0.3 0.3 2.44 2.29 1.55]*^T^* and ***x****_g_*_.2_^(0)^ = [0.41 0.59 0.3 0.3 2.35 2.61 1.23]*^T^* of both BLCs have been optimized (at the EM model level) using the algorithm of Section 2.4.1. The final designs ***x****_g_*_.1_^*^ = [0.35 0.53 0.42 0.4 2.4 2.25 1.52]*^T^* and ***x****_g_*_.2_^*^ = [0.38 0.54 0.23 0.41 2.63 2.68 1.27]*^T^* have been obtained after seven and nine iterations of (2), respectively. Magnitude and phase responses of both BLCs at ***x****_g.k_*^(0)^ and ***x***_g.k_^*^ are shown in Figure 8. The optimized designs are characterized by small footprints of 9 mm × 6.7 mm = 60.2 mm^2^ for BLC_1_ and 8.6 mm × 6.9 mm = 58.8 mm^2^ for BLC_2_, which corresponds to 79% miniaturization compared to couplers based on conventional TL sections [29].

**Figure 9:**
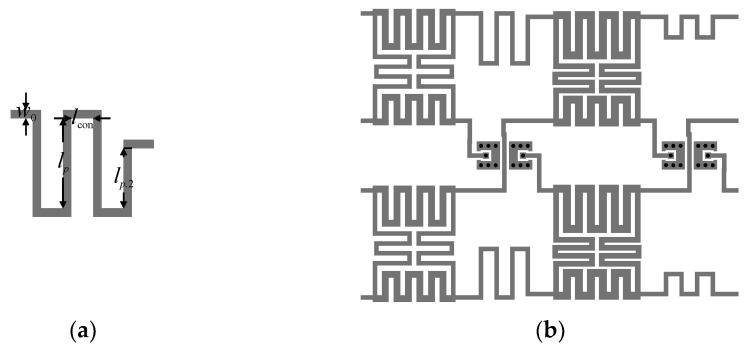
Geometry of (**a**) phase shifter with highlight on design parameters and (**b**) assembled structure of a compact Butler matrix.

The last step of sequential design involves optimization of phase shifters. The initial vector of adjustable electrical parameters is ***x****_e.p_*^(0)^ = [*β*_3_ + *β_c_ β*_3_ + *β_c_ β_c_ β_c_*]*^T^* = [106.2 106.2 61.2 61.2]*^T^* degrees (note that impedance of shifters is fixed to 50 Ω). The optimized values ***x***_e.p_^*^ = [105.9 106.7 60.9 61.3]*^T^* degrees are obtained through optimization of shifters embedded into the composite BM model as described in Section 3.2. The PS geometry is shown in Figure 9a. The adjustable parameter of *p*th structure (*p* = 1, 2, 3, 4) is *x_g.p_* = *l_p_*, whereas the relative dimensions that allow for maintaining geometrical consistency of the BM are *l_c_* = 0.2(*l*_0.cross_ − 4*w*_0_), *l_p_*_.2_ = *l_p_* − Δ*l*, and Δ*l* = *h*_BLC.2_ − h_BLC.1_ (here, *h*_BLC.*k*_ = 2*w*_0_ + *l*_2.*k*_ + 2*w*_2.*k*_ + *c*_2.*k*_ + 4*w*_1.*k*_ + 4*c*_1.*k*_ + *l*_3.*k*_, *k* = 1, 2); the width of meander lines is fixed to *w*_0_. Note that for *p* = 3 and *p* = 4, Δ*l* = 0 and thus *l_p_*_.2_ = *l_p_*. The optimized lengths *x_g_*_.1–4_ = *l*_1–4_ = {3.56, 3.6, 1.34, 1.36} mm of each meander section have been obtained sequentially as explained in Section 3.2.

**Figure 10:**
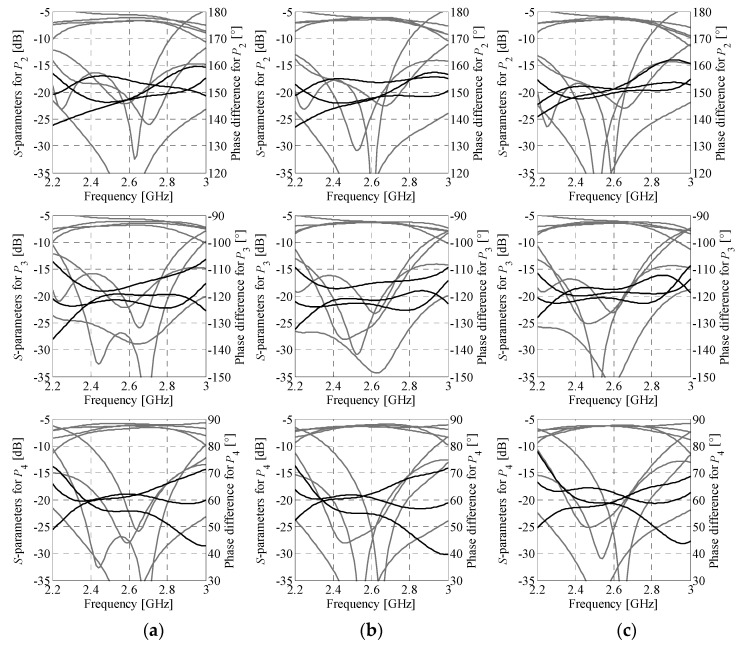
Butler matrix 1: structure characteristics at different stages of the design process. The responses are obtained from evaluations of the composite model ***R****_Be_* at (**a**) the initial design ***z***^(0)^, (**b**) the final design z^*^ (used as a starting point for fine-tuning), and (**c**) the optimized design y^*^ found based on simulations of the assembled BM model ***R****_Be_*. Each row shows responses of the structure “seen” from different input port (*P*_1…4_). Gray and black lines represent magnitude and phase responses, respectively.

The geometry of the assembled Butler matrix is shown in Figure 9b. The design parameters of the composite model ***R****_Bc_* used for optimization are ***z*** = [***x****_g_*_.1_ ***x****_g_*_.2_ *l*_1_ *l*_2_ *l*_3_ *l*_4_]*^T^*. The starting point is set to ***z***^(0)^ = [0.35 0.53 0.42 0.4 2.4 2.25 1.52 0.38 0.54 0.23 0.41 2.63 2.68 1.27 3.56 3.6 1.34 1.36]*^T^*. The optimal design z^*^ = [0.33 0.53 0.43 0.42 2.36 2.21 1.51 0.4 0.58 0.22 0.4 2.62 2.66 1.23 3.7 3.69 1.36 1.36]*^T^* has been found after six iterations of (2). It should be emphasized that, owing to the use of composite model for optimization of ***z***, each successful iteration of (2) required only two EM model evaluations of each BM component. The design z^*^ has been used as the initial point for fine-tuning of the matrix. The final design resulting from the tuning process of the ***R****_Be_* model y^*^ = [0.35 0.54 0.46 0.40 2.39 2.26 1.48 0.43 0.61 0.22 0.35 2.56 2.72 1.22 3.75 3.66 1.37 1.32]*^T^* has been obtained in eight iterations of (3). The dimensions of the optimized matrix are only 24 mm × 29 mm with overall footprint of 696 mm^2^. A comparison of the BM responses obtained through simulation of the ***R****_Be_* model at the designs ***z***^(0)^, z^*^, and y^*^ is shown in Figure 10.

The simulation results indicate that optimization of the composite model is important for improving transmission responses of the matrix, whereas fine-tuning provides correction of the output-port phase differences. At the final design y^*^, the ***R****_Be_* model response features in-band matching and isolation above the level of 15.5 dB. Furthermore, at the center frequency it offers insertion loss imbalance below 0.5 dB and phase shift errors below 2.6°. It should be noted that although the optimized design slightly violates the target values of (12)—defined in Section 3.2—this is justified as the objective function comprises a composition of design requirements, balanced by the user-defined weighting factors. Table 1 provides more information on performance of the matrix in terms of reflection *R*_BW_, isolation *I*_BW_, transmission imbalance Δ*M*_BW_, and phase imbalance Δ*P*_BW_ within 2.5 to 2.7 GHz band, as well as transmission Δ*M_f_*_0_ and phase Δ*P_f_*_0_ imbalances at the center frequency. The quantities *R*_BW_ and *I*_BW_ refer to the worst-case in-band performance (across all considered responses), whereas imbalance represents maximum difference between the considered groups of characteristics, either within the band of interest or at the center frequency.

The proposed design procedure has been benchmarked against the method where the fine-tuning step governed by algorithm (2) involves only evaluations of the ***R****_Be_* model [45]. For fair comparison, it is assumed that the design ***z***^(0)^—obtained through individual optimization of BM components—is used as a starting point for adjustment of the structure topology. The assumption seems justified considering that it follows the industry-wide divide-and-conquer strategy to design of complex circuits [27,36,53,54]. The tests have been performed on an Intel Xeon machine with 32 GB RAM. For the given machine, the average cost of EM simulation of BLC, PS, and assembled BM amounts to 4.5 min, 36 s, and 41 min, respectively. The results shown in Table 2 indicate that the proposed procedure is capable of yielding a design with competitive performance compared to the method that does not blend ***R****_Be_* and ***R****_Bc_* models, yet at a fraction of its computational cost (13.2 h for the presented approach vs. 91.6 h for conventional method). The number of model evaluations in Table 2 refers to all simulations of particular models required to find the final design.

### 4.2. Butler Matrix 2

The second design example is the Butler matrix structure featuring phase shifts Δ*θ*_1–4_ = {−20°, 160°, −110°, 70°}. The electrical properties of the matrix components required to obtain desired Δ*θ_j_* values are *β*_1_ = −65°, *β*_2_ = −40°, and *β*_3_ = −45° (cf. Section 3.1). The BM topology is shown in Figure 9b. The crossover dimensions remain the same as for the one used in Section 4.1. The initial dimensions of both BLCs ***x****_g_*_.1_^(0)^ = [0.41 0.59 0.3 0.3 2.31 2.48 1.39]*^T^* and ***x****_g_*_.2_^(0)^ = [0.59 0.72 0.3 0.3 1.5 2.79 0.54]*^T^* have been determined as described in Section 3.2 and Section 4.1 The optimized dimensions ***x****_g_*_.1_^*^ = [0.37 0.53 0.36 0.26 3.06 2.89 1.6]^T^ and ***x****_g_*_.2_^*^ = [0.57 0.67 0.33 0.34 1.48 2.95 0.58]*^T^* have been obtained after seven and nine iterations of (2), respectively.

**Figure 11:**
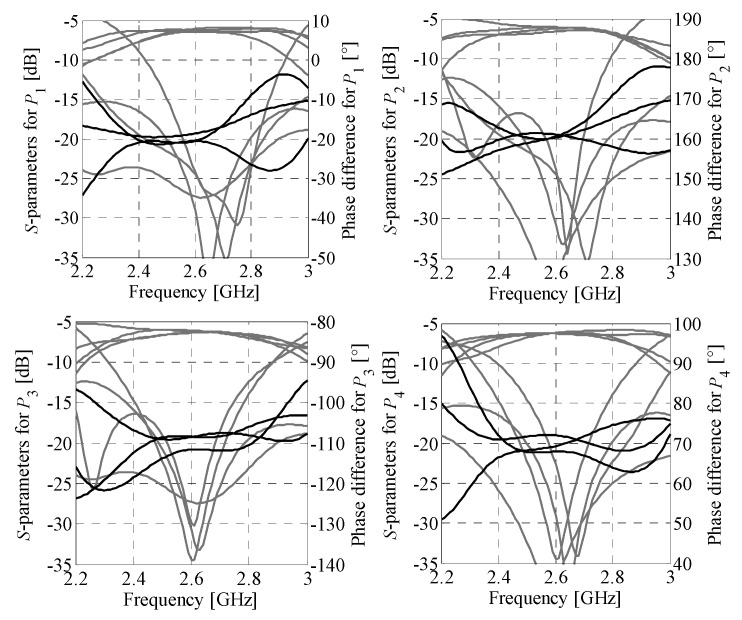
Butler matrix 2: EM-model-based magnitude (gray) and phase (black) responses of the optimized structure.

The initial design for Butler matrix optimization (composite model) is ***z***^(0)^ = [0.37 0.53 0.36 0.26 3.06 2.89 1.6 0.57 0.67 0.33 0.34 1.48 2.95 0.58 3.56 3.6 1.34 1.36]*^T^*. The optimized design (used as a starting point for fine-tuning of the BM) z^*^ = [0.36 0.52 0.38 0.26 3.09 2.89 1.59 0.58 0.69 0.33 0.34 1.49 2.95 0.59 3.57 3.6 1.35 1.37]*^T^* has been obtained after seven iterations of (2). The final design y^*^ = [0.35 0.51 0.42 0.25 3.11 2.90 1.56 0.64 0.73 0.34 0.27 1.53 2.95 0.52 3.57 3.56 1.39 1.36]*^T^* has been found after nine iterations of (2). The optimized BM is characterized by the dimensions of 25.9 mm × 29.6 mm and overall footprint of only 766.6 mm^2^. The response characteristics of the matrix are shown in Figure 11. The BM features in-band reflection below −14 dB and isolation higher than 20 dB, respectively. Moreover, at *f*_0_ the structure offers insertion loss imbalance below 0.55 dB and phase shift imbalance below ±2.5° w.r.t. the target values. It should be noted that the slightly worsened in-band reflection compared to the structure of Section 4.1 results from the narrow bandwidth of the BLC required for the realization of the −45° phase shift. The performance characteristics of the optimized structure are gathered in Table 3 (for explanation of used performance metrics, see Section 4.2).

### 4.3. Numerical and Experimental Validation

**Figure 12:**
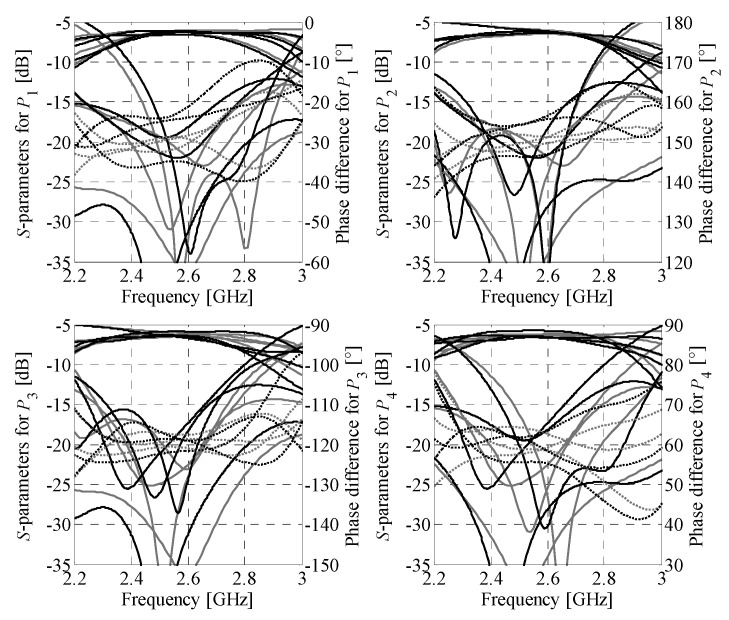
Butler matrix 1: comparison of the EM model responses obtained using CST Studio (gray) and Keysight ADS (black). Solid and dotted lines represent magnitude and phase responses, respectively.

**Figure 13:**
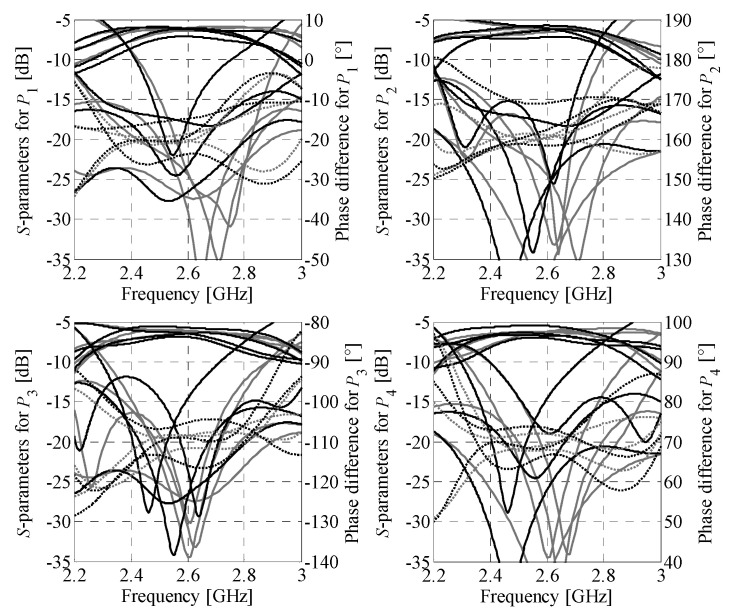
Butler matrix 2: comparison of the EM model responses obtained using CST Studio (gray) and Keysight ADS (black). Solid and dotted lines represent magnitude and phase responses, respectively.

Simulation-based validation of the optimization results has been performed through evaluation of the optimized designs using a solver based on the method of moments (Keysight ADS). A comparison of the performance characteristics obtained using CST Studio and Keysight ADS is shown in Figure 12 and Figure 13, respectively. The simulation results for BMs feature reflection below −12 dB and isolation above 13 dB within the bandwidth of interest. Regardless of the selected excitation port, the first design offers the insertion-loss imbalance below 0.9 dB and phase shift error below 8°. For the second matrix, the values are 1.5 dB and 9°, respectively. The obtained results indicate that the EM simulation results are valid.

**Figure 14:**
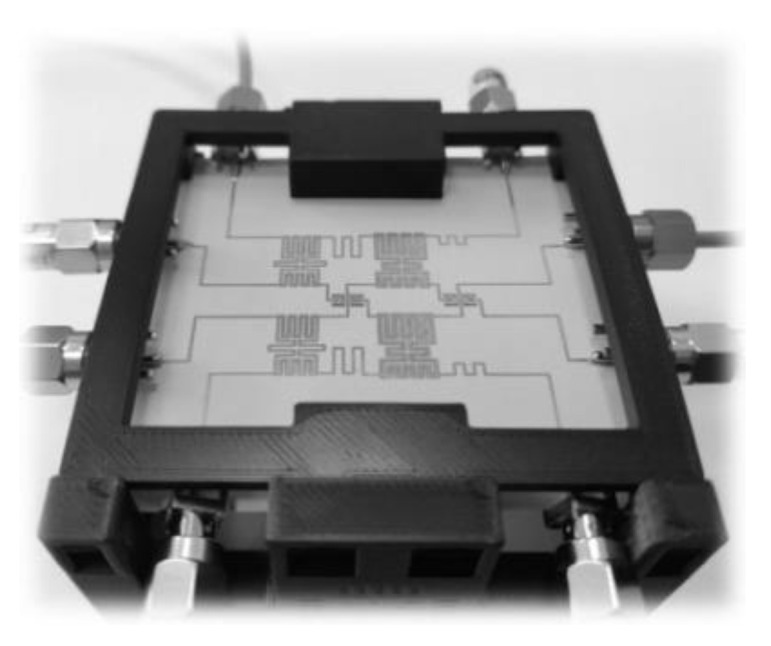
BM of Section 4.2: photograph of the fabricated prototype.

**Figure 15:**
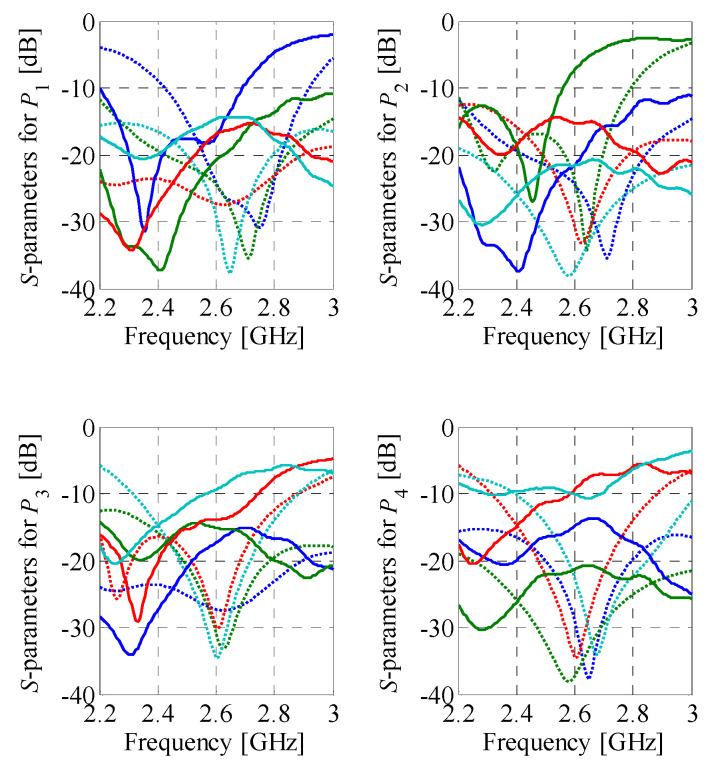
Butler matrix 2: comparison of measurements (––) with EM simulations (···) in terms of reflection/isolation. Here, the EM simulation model does not account for systematic errors.

**Figure 16:**
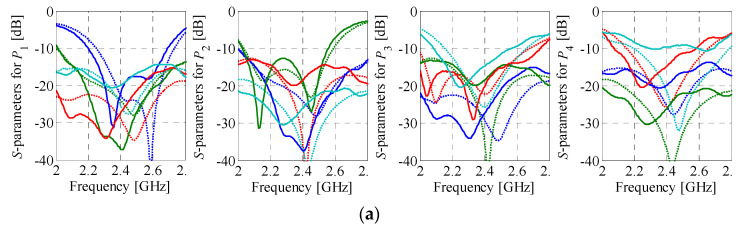

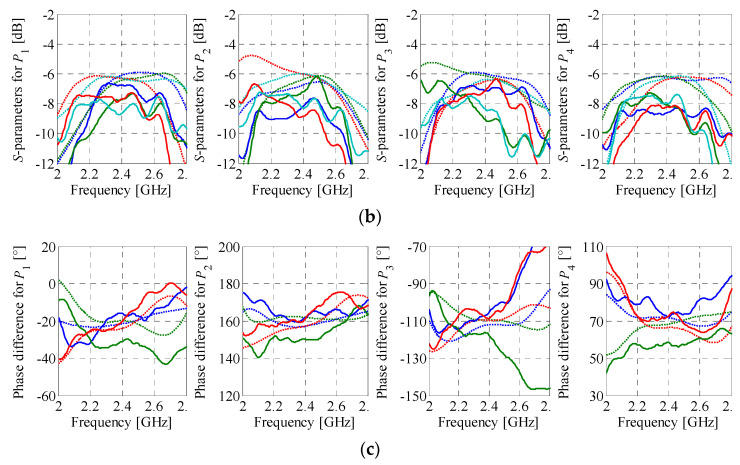
Measurements (––) vs. simulations (···) obtained using the EM model that account for the systematic errors: (**a**) reflection/isolation, (**b**) transmission, and (**c**) output-port phase differences.

For further verification of the results, the structure of Section 4.2 has been fabricated and measured. The photograph of a manufactured prototype is shown in Figure 14. To facilitate the measurement process, feed lines of the structure have been modified to make space for connectors. A comparison of reflection/isolation characteristics obtained from simulations and measurements is shown in Figure 15. The misalignment between the responses (most notably the frequency shift) is the consequence of the systematic error caused by differences between the relative permittivity and thickness of the substrate used for simulations and measurements. As can be seen from Figure 16, accounting for systematic errors significantly improves alignment between the responses. Other factors affecting the discrepancies include manufacturing tolerances, as well as assembly- (manual positioning/soldering of the connectors) and measurement-related errors (also resulting from tolerances of the matching loads) [55]. It should be emphasized that connectors alter electrical properties of the measured BM, which is mostly due to discontinuities at their interface with microstrip lines. The effect contributes to degradation of measured performance as compared to simulations. Nevertheless, the agreement between the simulations and measurements can be considered acceptable. A summary of the measured structure performance is provided in Table 4. In many cases, resemblance between EM simulations and measurements can be further increased by excluding the effects of the fixture (here, connectors along with introduced feeding lines) on the device under test [56]. Unfortunately, high sensitivity to precision of the assembly makes the method unsuitable for the discussed setup.

### 4.4. Comparison with Benchmark Structures

The optimized designs from Section 4.1 and Section 4.2 have been compared in terms of size and performance with other planar BMs from the literature [16,21,23,27,28,29,30]. Whenever possible (all benchmark designs except the one reported in [28]), the performance figures have been obtained based on the EM simulation results. The considered figures include magnitude imbalance Δ*M_f_*_0_ and phase shift Δ*P_f_*_0_ at the center frequency, as well as bandwidth BW. The latter is expressed in percent and calculated as a ratio of the difference between the upper and lower frequencies (i.e., the ones for which isolation is above 15 dB and reflection below −15 dB) to the specified *f*_0_. All of the considered figures represent the worst-case scenario for a series of analyses w.r.t. all input ports. For fair comparison of size, the dimensions of all matrices are expressed in terms of a guided wavelength *λ*_g_ (defined for the given center frequency and electrical parameters of the substrate used to implement the circuit). The results shown in Table 5 indicate that the designs obtained using the proposed procedure are characterized by competitive performance (with particular emphasis on unconventional output-port phase differences). Moreover, at roughly 10-fold smaller size w.r.t. conventional BM, the obtained designs outperform other structures in terms of miniaturization. It should be emphasized that compact dimensions, competitive magnitude/phase performance, and the center frequency of around 2.6 GHz make the optimized BMs of potential use for IoT devices interconnected through a 5G backbone [57].

## 5. Conclusions

In this work, a bottom-up framework for low-cost automated design of 4 × 4 Butler matrices with nonstandard output-port phase shifts has been presented. The technique involves sequential development of BM components followed by optimization of the composite structure representation and fine-tuning of the assembled matrix. Each design step is governed by optimization algorithm. The proposed approach has been demonstrated using two compact BM structures designed to operate at 2.6 GHz frequency. The first circuit, designed to provide phase shifts of {−30°, 150°, −120°, 60°}, offers simulation-based reflection below −15 dB within the 2.5–2.7 GHz band, along with low transmission and phase shift imbalance of 0.5 dB and ±2.5°. The second structure realizes phase shifts of {−20°, 160°, −110°, 70°} with in-band reflection below −14 dB, as well as phase and magnitude imbalance below 0.55 dB and ±2.5°, respectively.

The computational cost of BM design using the proposed strategy is over 80% lower compared to more conventional approach that does not exploit composite models. The structures have been compared against state-of-the-art BMs from the literature in terms of performance and size. With the footprints of only 696 and 767 mm^2^, the optimized circuits outclassed BMs from the literature in terms of size reduction while providing competitive performance. The unique feature of the presented designs—which makes them of potential use for IoT systems interconnected through the 5G communication network—is that they address requirements concerning both size reduction and enhanced performance, whereas the benchmark designs address only one of these figures at a time. The measurements of the fabricated BM prototype are also provided.

Future work will focus on increasing the flexibility of BMs in terms of attainable output-port phase shift as well as development of design methods that support algorithm-driven integration of feeding networks and radiators into a complete antenna array.

## Figures and Tables

**Table 1:** Butler matrix 1: performance figures of the optimized structure (EM simulation).

Excitation	Port *P*_1_	Port *P*_2_	Port *P*_3_	Port *P*_4_
*R*_BW_ (dB)	−21.2	−15.5	−17.3	−16.1
*I*_BW_ (dB)	18.4	17.6	17.6	18.1
Δ*M*_BW_ (dB)	0.79	0.75	0.74	0.45
Δ*M_f_*_0_ (dB)	0.51	0.45	0.41	0.14
Target phase (°)	−30	150	−120	60
Δ*P*_BW_ (°)	±4.9	±4.7	±4.0	±4.3
Δ*P_f_*_0_ (°)	±1.6	±1.3	±2.5	±2.6

**Table 2:** Butler matrix 1: numerical benchmark of the proposed design framework.

Method	EM Model Type	No. of Model Evaluations	Cost		Total Cost		Performance Figures *		
			(*R_Be_*)	(h)	(*R_Be_*)	(h)	*R_BW_* (dB)	Δ*M_f0_* (dB)	Δ*P_f0_* (°)
Direct	*R_Be_*	134	134	91.6	134	91.6	−15.2	0.59	±3.2
This work	BLC ^#^	60	6.6	4.5	19.4	13.2	−15.5	0.51	±2.6
	PS ^#^	120	1.8	1.2					
	*R_Be_*	11	11	7.5					

* Worst-case performance obtained for excitation of the BM using ports *P*_1–4_; ^#^ Different subcomponents of the BM are represented using the same EM model.

**Table 3:** Butler matrix 2: performance figures of the optimized structure (EM simulation).

Excitation	Port *P*_1_	Port *P*_2_	Port *P*_3_	Port *P*_4_
*R*_BW_ (dB)	−14.9	−17.1	−18.3	−14.5
*I*_BW_ (dB)	20.1	20.7	20.7	20.1
Δ*M*_BW_ (dB)	0.68	0.93	0.68	0.50
Δ*M_f_*_0_ (dB)	0.38	0.52	0.28	0.10
Target phase (°)	−30	150	−120	60
Δ*P*_BW_ (°)	±3.6	±4.3	±3.8	±3.1
Δ*P_f_*_0_ (°)	±1.3	±1.0	±1.6	±2.1

**Table 4:** Butler matrix 2: measured performance figures of the fabricated prototype.

Excitation *	Port *P*_1_	Port *P*_2_	Port *P*_3_	Port *P*_4_
*R*_BW_ (dB)	−17.6	−10.8	−14.5	−9.1
*I*_BW_ (dB)	15.8	14.5	10.3	10.9
Δ*M*_BW_ (dB)	2.01	2.75	3.99	1.05
Δ*M_f_*_0_ (dB)	1.34	1.91	2.59	0.85
Target phase (°)	−30	150	−120	60
Δ*P*_BW_ (°)	±14.3	±9.06	±18.4	±9.23
Δ*P_f_*_0_ (°)	±7.48	±7.83	±10.9	±8.79

* Due to frequency shift, the measured values are obtained for *f*_0_ = 2.45 GHz and BW from 2.35 to 2.55 GHz.

**Table 5:** Compact BM: comparison with state-of-the-art structures from the literature.

Designs		Performance Figures *				Size		Relative Size (%) ^$^
	*f*_0_ (GHz)	BW ^&^ (%)	Δ*M_f_*_0_ (dB)	Δ*θ*_1–4_ (°)	Δ*P_f_*_0_ (°)	Dimensions (mm × mm)	Dimensions (*λ_g_* × *λ_g_*)	
Conventional [21]	1.0	7.0	0.60	−45, 135, −135, 45	±4.0	233 × 191	1.29 × 1.06	100
[23]	5.8	7.3	0.45	−30, 150, −120, 60	±3.8	71.4 × 119	1.89 × 3.15	437
[27]	6.0	7.2	0.40	−45, 135, −135, 45	±0.9	58.9 × 57.9	1.96 × 1.93	279
[16]	28	3.2	4.70	−45, 135, −135, 45	±16	16.8 × 14.9	N/A ^#^	N/A ^#^
[28] ^!^	1.8	5.5	2.40	−45, 135, −135, 45	±5.9	99.5 × 127	0.82 × 1.04	62.8
[30]	1.0	5.9	2.30	−45, 135, −135, 45	±1.8	164 × 121	1.00 × 0.74	54.1
[29]	1.0	N/A	1.20	−45, 135, −135, 45	±1.0	87.8 × 82.4	0.49 × 0.46	16.3
This work (BM_1_)	2.6	8.4	0.51	−30, 150, −120, 60	±2.6	24.0 × 29.0	0.34 × 0.42	10.5
This work (BM_2_)	2.6	7.6	0.52	−20, 160, −110, 70	±2.1	25.9 × 29.6	0.37 × 0.42	11.6

* Worst-case performance obtained for excitation of the BM using ports *P*_1–4_; ^#^ Multilayer structure implemented using substrates with different electrical parameters; ^$^ With respect to conventional Butler matrix; ^!^ Simulation results not available—data obtained from measurements; ^&^ Calculated for reflection below −15 dB and isolation above 15 dB.

## Data Availability

Not applicable.
